# Spinal Epidural Abscess Caused by Campylobacter jejuni Without Gastrointestinal Symptoms

**DOI:** 10.7759/cureus.68408

**Published:** 2024-09-01

**Authors:** Hiroaki Yabe, Ryoichi Inoue, Ryo Yanai, Shinichiro Nishimi

**Affiliations:** 1 Division of Rheumatology, Department of Medicine, Showa University School of Medicine, Tokyo, JPN; 2 Department of Internal Medicine, The Ohio State University Wexner Medical Center, Columbus, USA

**Keywords:** immunosuppressant, spinal mri, mri contrast, lower back pain (lbp), campylobacter jejuni, spinal epidural abscess

## Abstract

*Campylobacter jejuni* (*C. jejuni*) is a gram-negative bacterium known to cause gastroenteritis with fever, abdominal pain, and bloody diarrhea. Although *Campylobacter* bacteremia is reported in patients with gastroenteritis, localized abscess formation, particularly spinal epidural abscess (SEA), is extremely rare and can easily be missed. Herein, we report a case of a 54-year-old immunocompromised female presenting with severe back pain without gastrointestinal symptoms, who was ultimately diagnosed with an L5/S1 SEA due to *C.jejuni*, requiring laminectomy and drainage. As far as we know, this is the second reported case of SEA due to *C. jejuni* without any preceding gastrointestinal symptoms. This case highlights the critical importance of performing a contrasted MRI for the early and accurate diagnosis of SEA.

## Introduction

*Campylobacter jejuni (C. jejuni)* is a spiral-shaped gram-negative bacterium that grows well in microaerophilic and capnophilic conditions and at the temperature range between 30 ℃ and 47 ℃ [1]. While the most common presentation is self-limiting gastroenteritis after ingestion of raw or undercooked meat, it can cause extra-intestinal conditions, including septic arthritis, skin and soft tissue infection, intraabdominal infection, and endocarditis [2,3]. It has also been reported that *Campylobacter* enteritis causes *Campylobacter* bacteremia in less than 1% of individuals and is more common in immunocompromised patients [4]. There are only a few cases reporting spinal epidural abscesses due to *C. jejuni *[5,6]. In this case, we report an epidural abscess caused by *C. jejuni* without any preceding gastrointestinal symptoms and highlight the importance of contrast MRI for an earlier diagnosis of SEA.

## Case presentation

**Figure 1:**
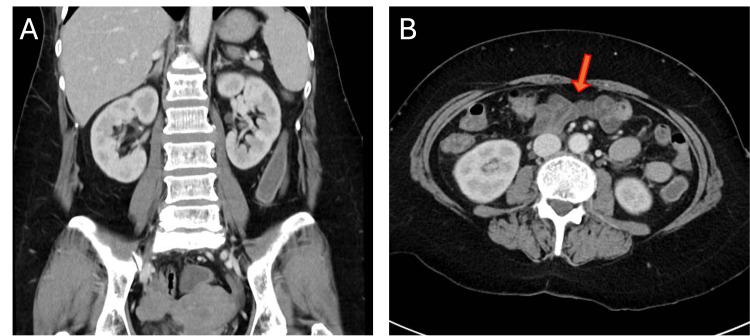
CT of the abdomen with contrast at her first presentation A: On the sagittal plane of the contrast-enhanced computed tomography (CECT), there is no evident contrast enhancement surrounding the psoas muscles or perinephric area. Although a compression fracture at L5 is observed, there are no surrounding contrast-enhancing lesions. B: In the axial plane of the contrast-enhanced CT, fluid retention is noted within the small intestine, and no other significant findings were observed.

**Figure 2:**
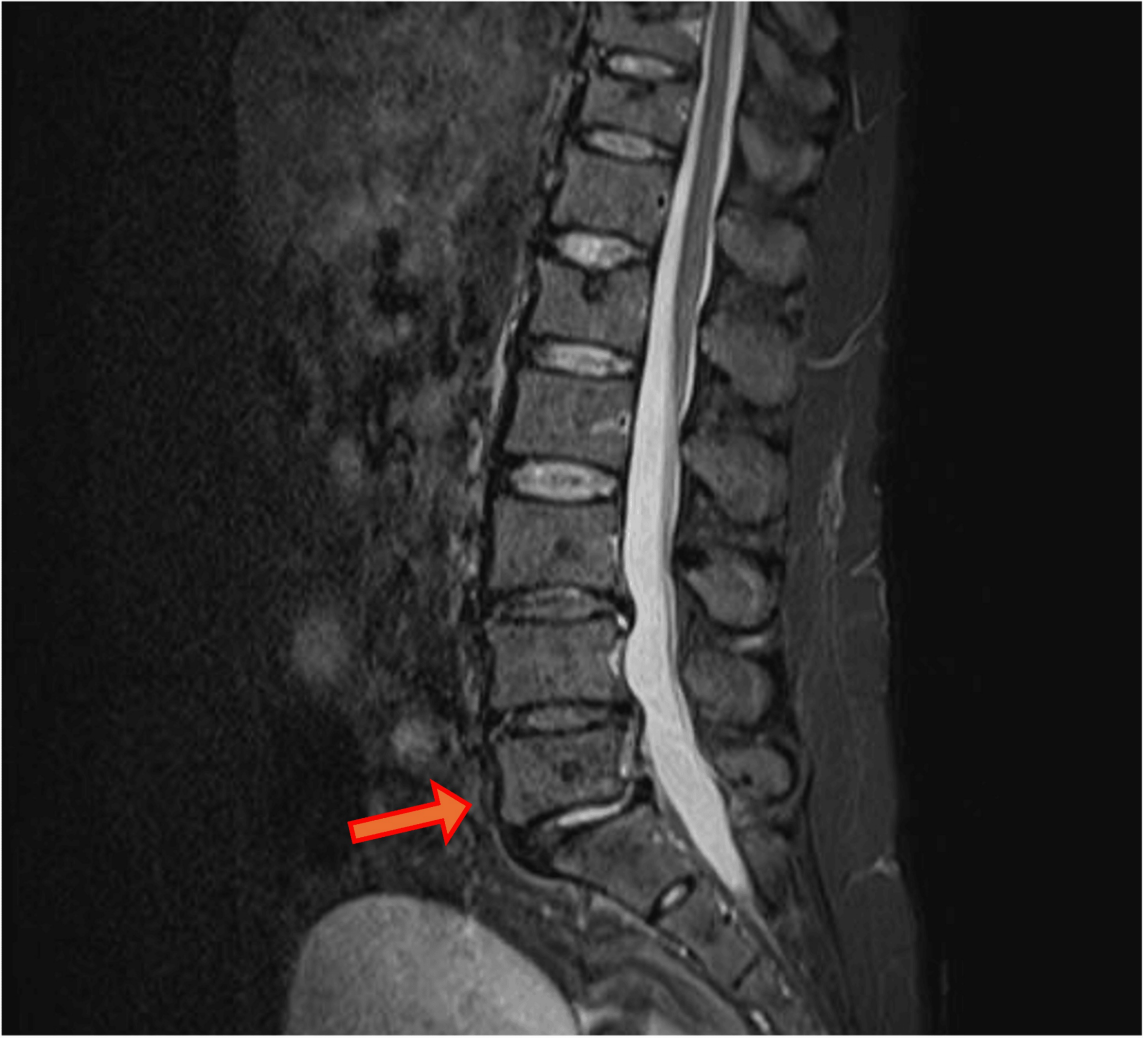
MRI STIR at the patient's first presentation In MRI STIR, compression fractures at L1 and spondylolisthesis at L5 (red arrow) are observed. No obvious abscesses surrounding the vertebral bodies are noted. STIR: short tau inversion recovery

A 54-year-old woman presented with fever and lower back pain for 2 days to the Emergency Department at Showa University Hospital, Japan. She had systemic lupus erythematosus for 15 years, and her current medications were prednisolone 2 mg, tacrolimus 3 mg, mycophenolate mofetil 1500 mg, and hydroxychloroquine 200/400 mg. After eating chicken seven days prior, the patient started experiencing back pain that gradually worsened over three days to the point where she became immobile due to pain. She denied any gastrointestinal symptoms, including abdominal pain, nausea, vomiting, diarrhea, or bloody stool. Her vital signs were within normal ranges except for a high fever (39.4 ℃). There were no abnormal findings on abdominal examination or costovertebral angle tenderness. The neurologic examination was unremarkable. Laboratory data showed elevated white blood cells (WBC, 11,600/μL) and C-reactive protein (CRP, 22.57 mg/dL) (Table 1). The CT of the abdomen with contrast was unremarkable except for fluid retention at the transverse colon and sigmoid colon (Figure 1). Short tau inversion recovery (STIR) MRI revealed compression at the L1 vertebra and slippage at L5 without a high-density region. At this point, our team could not identify any probable abscess based on the T2-weighted, STIR, and diffusion-weighted imaging (DWI) MRI (Figure 2). Since no infectious source was detected, we considered a compression fracture and concurrent bacteremia of an unknown source, and the patient was admitted for treatment and further assessment.

**Table 1:** Laboratory data at the patient's first presentation ds-DNA: double-stranded deoxyribonucleic acid

Laboratory investigations	Results	Normal range or result
Leucocyte count	11,600 /μL	4,000 - 9,000/μL
Neutrophil	87.90%	45.0 - 70.0 %
Lymphocyte	6.20%	30.0 - 45.0 %
Erythrocyte count	3.92 × 1,000,000/μL	3.8 - 5.1 × 1,000,000/μL
Hemoglobin	10.4 g/dL	12.0 - 16.5 g/dL
Hematocrit	32.80%	35.0 - 45.0 %
Platelet	16.5 × 10,000/μL	15.0 - 35.0 × 10,000/μL
Creatinine	0.61 mg/dL	0.40 - 0.70 mg/dL
Urea nitrogen	21.0 mg/dL	8.0 - 22.0 mg/dL
Aspartate transaminase	42 U/L	13 - 33 U/L
Alanine transaminase	19 U/L	6 - 27 U/L
C-reactive protein	22.57 mg/dL	< 0.30 mg/dL
Ferritin	125 ng/mL	30 - 200 ng/mL
Immunoglobins G	893 mg/dL	870 - 1,700 mg/dL
Complement 3	134.0 mg/dL	65.0 - 135.0 mg/dL
Complement 4	35.2 mg/dL	13.0 - 35.0 mg/dL
Anti-ds DNA titers	11 IU	< 25 IU

After the two sets of blood cultures were taken, the patient was empirically started on intravenous piperacillin/tazobactam 4.5 g three times a day for eight days and intravenous azithromycin 500 mg once a day for three days. In a few days, the two sets of anaerobic blood cultures turned positive for *Campylobacter jejuni*. The antibiotics were deescalated to oral levofloxacin 500 mg once a day. The fever improved on the second day of her admission. She received nonsteroid anti-inflammatory drugs for the lower back pain and her pain was relieved, which improved enough for her to manage to walk. After 15 days of antibiotic treatment, her labs showed improvement in her WBC (3,300/μL) and CRP (2.53mg/dL), and she was discharged.

**Figure 3:**
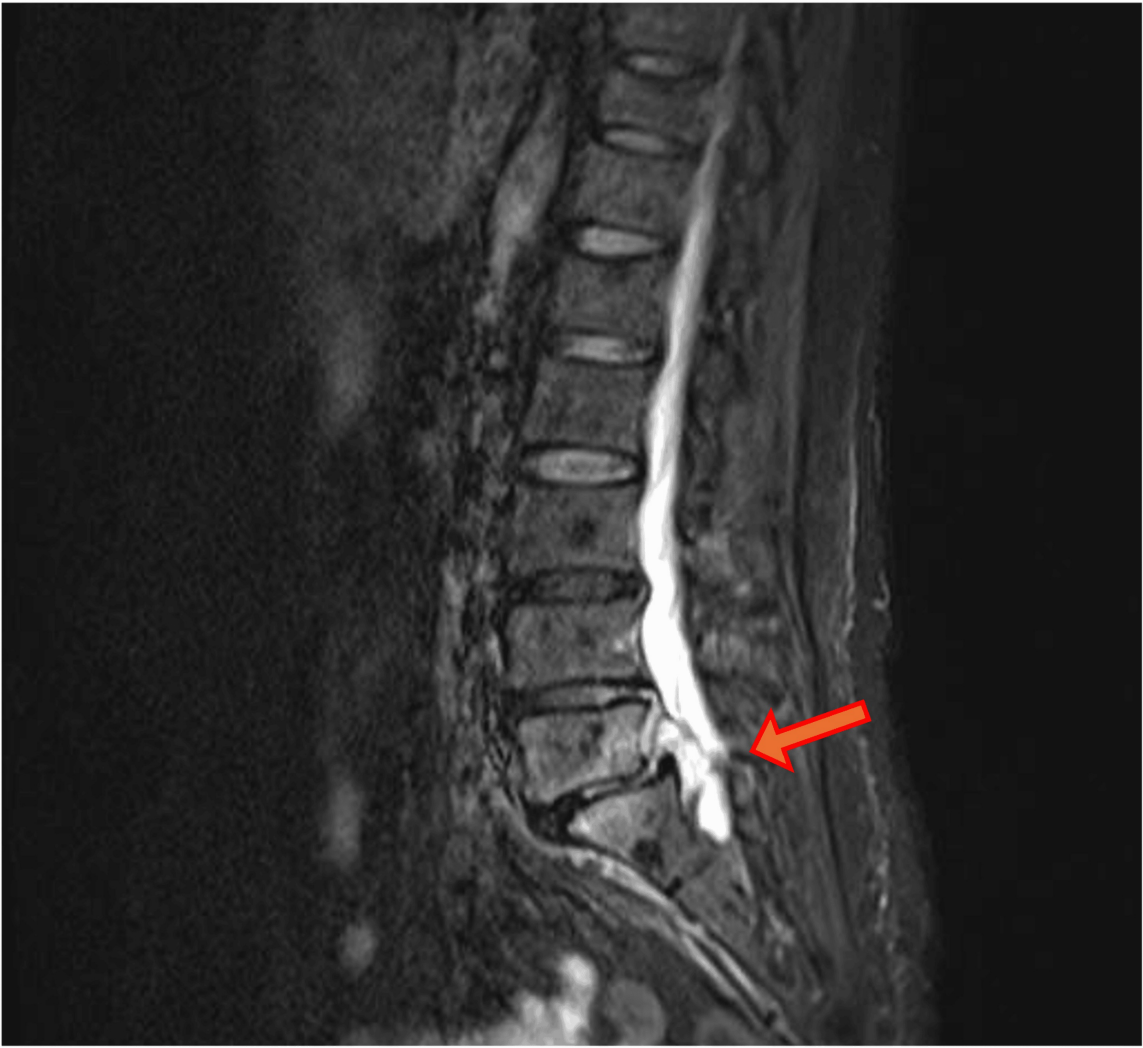
The MRI STIR at her second admission On the MRI STIR at the second admission, discitis at L5/S1 and a left-sided epidural abscess at S1 (red arrow) were observed. STIR: short tau inversion recovery

However, she again developed back pain two days after discharge and became immobile due to pain. Her CRP (12.36 mg/dL) was elevated while her WBC (3300/μL) was unchanged. The second STIR MRI revealed L5/S1 intervertebral discitis and S1 epidural abscess (Figure 3). We restarted intravenous piperacillin/tazobactam 4.5 g three times a day. But her symptoms did not disappear. After consulting orthopedics, she underwent a laminectomy and abscess drainage on day 31 since her initial presentation. A culture was taken but did not yield any growth. We continued to use piperacillin/tazobactam until the surgery. After drainage, all laboratory data and images were observed again, and she was discharged on day 61 since her initial admission.

## Discussion

This case recounted a rare presentation of *C. jejuni* with SEA without preceding gastrointestinal symptoms. It also highlights the importance of Gadolinium-enhanced MRI for the diagnosis of SEA. Our patient had a recurrent presentation, which was possibly due to the initial failure to detect SEA with contrast CT and non-contrast MRI.

Our patient developed SEA due to *C. jejuni* without any gastrointestinal symptoms. According to a PubMed search in August 2024 (“Spinal epidural abscess” and “Campylobacter jejuni”), there were only two case reports of SEA due to *C. jejuni*. Karina Frahm Kirk et al. reported epidural abscesses followed by osteomyelitis due to *C.jejuni* with GI symptoms [5]. Kamm CP et al. reported an epidural abscess by *C. jejuni*, causing polyradiculoneuropathy, without GI symptoms [6]. Given the rarity of SEA development by *C.jejuni*, we hypothesized that this patient's immunocompromised condition due to the treatment for SLE was one of the factors. Also, abnormality of the vertebral column could be a risk factor [7]. These factors promoted *C. jejuni* bacteremia, contributing to the development of SEA. 

In our patient, the initial blood culture was positive for *C. jejuni*, but the culture of the aspirated abscess was negative. This is probably because of a technical issue regarding the abscess aspiration for the culture. Once the abscess is opened, *Campylobacter* does not grow adequately in oxygen-exposed conditions. PCR examination could have allowed for identification instead of the culture test, but was not available at our facility.

A SEA was not initially visible with contrast CT and non-contrast MRI of the spine, which led us to omit contrast MRI for further investigation. Given our delay of care for her SEA, it should be noted that a Gd-enhanced MRI should be performed to completely rule out SEA, especially in patients with high pretest probability. Although no studies compare the sensitivity and specificity of CT and MRI for spinal epidural abscess, according to the sensitivity of imaging studies of diagnosis of psoas abscess, plain CT has 33% sensitivity, enhanced CT has 50%, and plain MRI has 50% [7,8]. This data underlines the importance of enhanced MRI even if the other image is negative when abscesses are considered.

**Figure 4:**
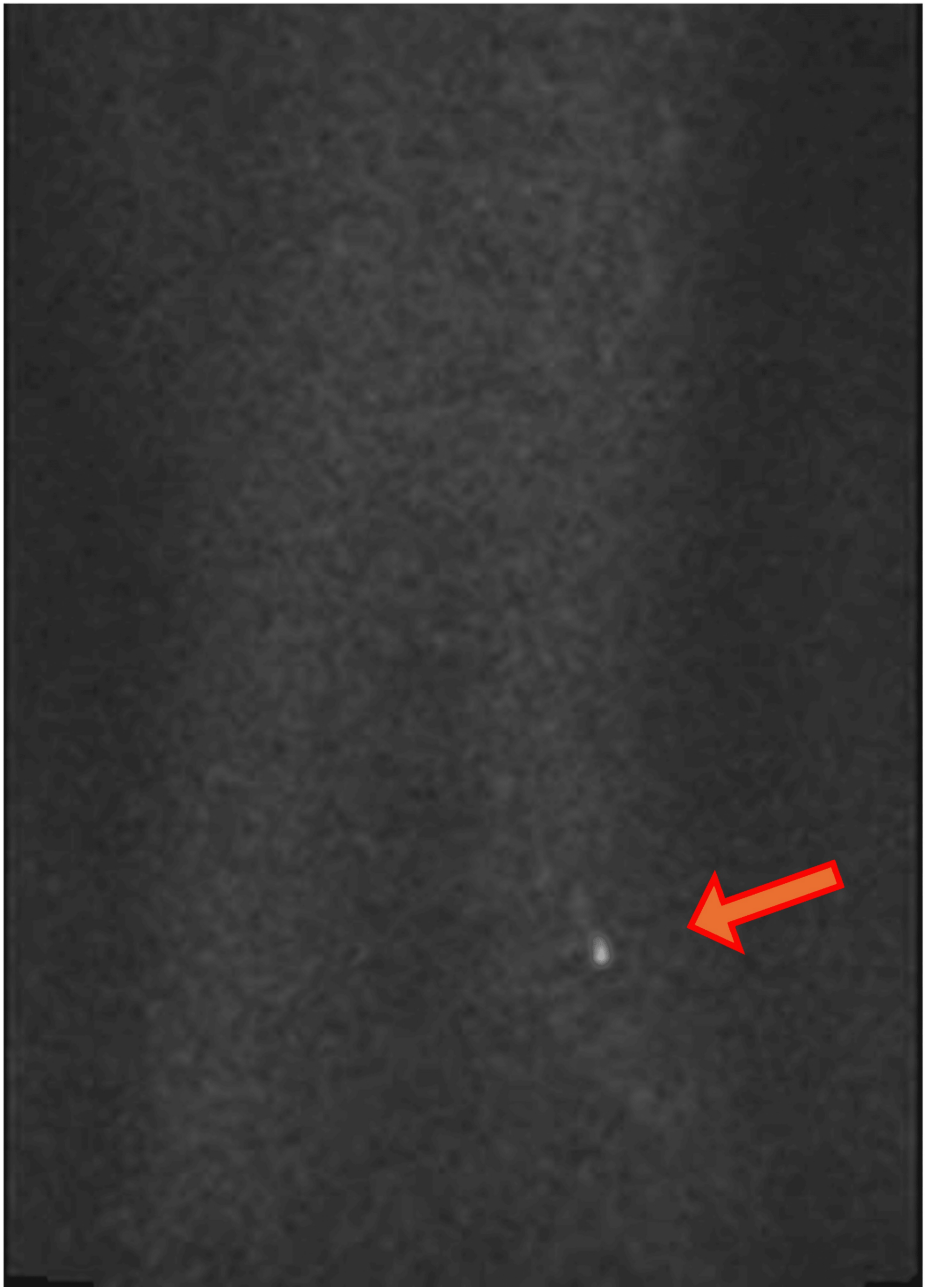
The DWI MRI at her first presentation This is a sagittal section taken with a DWI MRI at the first hospital admission. Although a small area of high signal (red arrow) was observed around L5/S1, clinical relevance was not appreciated. DWI: diffusion-weighted imaging

DWI-MRI is reported to be useful for detecting SEA [9]. In retrospect, our patient demonstrated small DWI-high signal intensity in the L5/S1 level of the spine at the first presentation, which might have indicated SEA (Figure 4). Furthermore, the apparent diffusion coefficient (ADC) might increase the detection rate of spinal infections compared to DWI alone [10]. Given the potential utility of non-contrast MRI, we could have detected SEA earlier at that point, especially with a high enough clinical suspicion leading to earlier surgical management. In addition to these benefits, contrast MRI should be performed if there is a high pretest probability of abscess.

## Conclusions

*C. jejuni* can cause SEA without preceding gastrointestinal symptoms. In the appropriate clinical setting (immunocompromised, abnormality of the vertebral column, and constitutional symptoms), clinicians should have a high suspicion of SEA and consider contrast MRI even when contrast CT and non-contrast MRI fail to detect it.
